# Sample handling of gastric tissue and *O*-glycan alterations in paired gastric cancer and non-tumorigenic tissues

**DOI:** 10.1038/s41598-017-18299-6

**Published:** 2018-01-10

**Authors:** Barbara Adamczyk, Chunsheng Jin, Karol Polom, Pedro Muñoz, Miguel A. Rojas- Macias, David Zeeberg, Mats Borén, Franco Roviello, Niclas G. Karlsson

**Affiliations:** 1 Department of Medical Biochemistry and Cell Biology, Institute of Biomedicine, Sahlgrenska Academy, University of Gothenburg, Gothenburg, Sweden; 2 Department General Surgery and Surgical Oncology, University of Siena, Siena, Italy; 3 Department of Surgical Oncology, Medical University of Gdansk, Gdansk, Poland; 4 Department of Biochemistry and Molecular Biology III, School of Medicine, Complutense University of Madrid, Madrid, Spain; 5 Denator AB, Uppsala Science Park, Uppsala, Sweden; 6 Department of Medicine, Surgery and Neurosciences, University of Siena, Siena, Italy

## Abstract

Sample collection, handling and storage are the most critical steps for ensuring the highest preservation of specimens. Pre-analytical variability can influence the results as protein signatures alter rapidly after tissue excision or during long-term storage. Hence, we evaluated current state-of-the-art biobank preservation methods from a glycomics perspective and analyzed *O*-glycan alterations occurring in the gastric cancer tissues. Paired tumor and adjacent normal tissue samples were obtained from six patients undergoing gastric cancer surgery. Collected samples (n = 24) were either snap-frozen or heat stabilized and then homogenized. Glycans were released from extracted glycoproteins and analyzed by LC-MS/MS. In total, the relative abundance of 83 *O*-glycans and 17 derived structural features were used for comparison. There was no statistically significant difference found in variables between snap frozen and heat-stabilized samples, which indicated the two preservation methods were comparable. The data also showed significant changes between normal and cancerous tissue. In addition to a shift from high sialylation in the cancer area towards blood group ABO in the normal area, we also detected that the LacdiNAc epitope (*N*,*N*’-diacetyllactosamine) was significantly decreased in cancer samples. The *O*-glycan alterations that are presented here may provide predictive power for the detection and prognosis of gastric cancer.

## Introduction

Glycomics is an emerging scientific discipline that seeks to understand the structure and function of glycans in biological systems. Glycan biosynthesis is a post-translational modification (PTM) and is not directly template-driven. Therefore, multiple factors can impact the glycosylation process, including glycosyltransferases accessibility to glycosylation modification sites, availability of sugar-nucleotide precursors, transport rates of glycoproteins from the ER to Golgi, enzyme levels or localization of enzymes that can compete for the same glycan substrates^1,2^. Many physiological changes are mediated by PTMs and cannot be predicted from the nucleic acid sequence. Aberrant glycosylation has been reported in many diseases such as rheumatoid arthritis, congenital disorders of glycosylation, and cancer, to name but a few^3–6^. However, due to the complexities in glycan biosynthesis, there are significant analytical challenges associated with the analysis of glycans in clinical samples^7^.

Sampling procedure is often the first but often the most critical step for ensuring the highest preservation of samples and the best quality for pre-clinical, translational or clinical projects. Good sampling and sample preparation influence the robustness and performance of analytical methods^8^. However, these steps are often overlooked, and most of the emphasis is placed on the development of an analytical method. When tissue sample is removed from its native environment, several alterations at the molecular level occur due to absence of vascular perfusion, ischemia, hypoxia, accumulation of cellular waste, absence of electrolytes and temperature changes^9^. In addition to stress associated with tissue excision, variability during collection procedures, delay in sample freezing, degradation following sample collection or freeze/thaw cycles can influence quality and quantity of proteins/glycoproteins in these matrices. Universal approach for sample preservation involves application of protease inhibitors and cold temperature/freezing.

Another preservation technology has been developed combining convective heating under controlled pressure that stop degradation and alterations immediately and permanently^10^. Benefits of using this platform have been demonstrated for small molecules, lipids, endogenous peptides and phosphoproteins^11–14^. Several studies have been conducted concerning the stability of proteins and phosphoproteins post excision^15,16^ but to the best of our knowledge, stability of glycoproteins has not previously been examined. Some of the glycan residues may be prone to degradation, for example desialylation, desulfation and de-*O*-acetylation. The problem associated with sialic acid and its labile nature has been addressed by developing chemical derivatization approaches^17–19^. However, analysis of underivatized glycans is still commonly used in glycoproteomic and glycomic studies^20–22^.

Elucidating the role of proteins and their post-translational modifications is crucial to understand biological mechanisms and define potential biomarkers. Exploiting the differences in glycan repertoires between cancerous and normal tissues provides opportunities to discover new targets for personalized cancer diagnosis and treatment. Discovery of these cancer-associated modifications of glycans on the glycoproteins may also improve on the specificity of existing cancer biomarkers. Gastric cancer (GC) is a major worldwide cancer burden with almost 1 million cases in 2012 and an annual mortality rate of approximately 723,000^23^. The treatment is most effective in the initial stage of the disease and hence early detection could reduce mortality. Although screening methods currently used in the clinic for GC improved in the recent past, they are still expensive, invasive and/or uncomfortable for the patients. Several serological assays are used in GC diagnosis and prognosis, e.g., CEA, CA19-9, CA72-4 and CA50 but they often lack sensitivity and specificity^24,25^. Thus, there is still an unmet need for the development of accurate biomarkers for the early detection and follow-up of gastric cancer patients. Alterations of glycan structures can influence their interactions with glycan-binding proteins and as a result affect cellular processes, such as tumor progression, metastasis and recognition by the immune system^26^. Hence, adding information on glycan changes to screening of protein levels is an attractive approach.

With glycomics being a relatively young area of research, we are just starting to learn how to utilize available biobanks for glycomic analysis. Biobanks and archived data sets have become crucial resources for many studies relying on sample collections with associated clinical and pathological data. Many population-based and disease-based biobanks have been formed, and more are under development^27,28^. These large-scale repositories will play an important role for investigating human diseases by omics technologies. The suitability of biological samples collected in the past and currently stored in biobanks is unknown. In proteomics, there has been concern that variability related to pre-analytical delays can impact the results as protein signatures alter rapidly when tissues experience the traumatic event of excision or samples are stored over prolonged periods. Hence, we evaluated current state-of-the-art biobank preservation methods from a glycomics perspective. We examined the effect of snap freezing versus heat-stabilization technology on gastric *O*-glycans and *O*-glycan alterations occurring in the gastric cancer tissues in comparison to adjacent normal mucosa. By further defining the gastric cancer specific glycosylation utilizing the *O*-linked technology platform, more accurate monitoring of the gastric carcinogenesis processes can be achieved.

## Results

### Method optimization

The main goal of sample homogenization is to solubilize as many proteins as possible from a tissue for the full representation of the proteome (glycoproteome). Aiming to select the best conditions for efficient extraction of proteins from tissue samples, we evaluated **a)** manual vs. semi-automatic homogenization **b)** solubilisation buffers with two different detergents – the ionic SDS and zwitterionic CHAPS and **c)** whether sonication was an essential step to solubilize tissue. Due to a very precious clinical material, we have optimized the protocol on porcine gastric tissue. Three different tissue homogenization and extraction steps were optimized using either the Precellys 24, Ultra Turrax T10 homogenizer with dispersing element S10N-5G and a sonicator probe. Efficient tissue homogenization is a prerequisite for optimal protein extraction and is often performed manually, one sample at a time. However, this approach is very tedious and often prone to errors and diminished reproducibility. To increase speed and reproducibility we tested homogenization tubes with ceramic beads for automated tissue homogenization. There are several types of homogenization tubes and beads available from instrument manufactures. The tubes, the time and power settings needed for homogenization depend on the tissue type but since gastric tissue is rather a tough tissue we decided to use 2 mL reinforced propylene tubes in combination with 3-mm ceramic beads as these have been showed previously to withstand the high power setting (5000) during 2–3 × 20 sec cycles on Precellys system^29,30^. These were evaluated previously for lipid extraction^29^. We did not observe any significant differences between manual and semi-automatic homogenization (data not shown).

**Figure 1:**
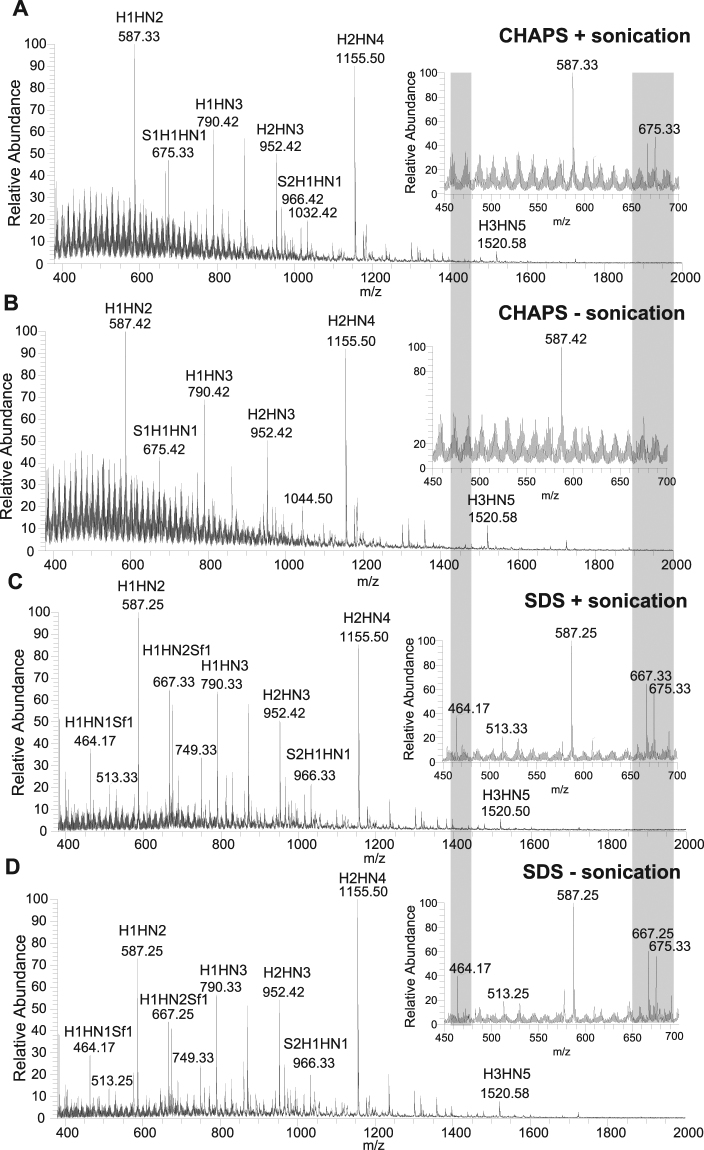
Comparison of LC-MS spectra of *O*-linked glycans released from porcine gastric tissue 1) CHAPS + sonication, 2) CHAPS without sonication, 3) SDS with sonication 4) SDS without sonication. (**A**) LC-ESI-MS profiles showing a full *m/z* range (380–2000), panels on the right illustrate an enlarged region of *m/z* 450–700, region that differ between the samples is highlighted in grey. Major glycan peaks are labeled with *m/z* values and compositions (H, Hexose; HN, *N*-acetylhexosamine; S, Sialic acid (*N*-acetylneuraminic acid); Sf, Sulfate).

Urea-based buffers are widely used for sample homogenization and has been shown to provide higher protein yields than non-urea buffers^31^. Therefore, we decided to use urea-based buffer and optimize concentration of detergents to facilitate recovery of *O*-glycans from gastric tissue. We have tested SDS and CHAPS. Figure 1 represents the full range LC-MS spectral profiles as well as enlarged area of lower mass range. The resulting data showed very similar relative abundance of *O*-glycan structures. However, we found that samples solubilized in SDS (Fig. 1C,D) provided lower background than samples solubilized in CHAPS (Fig. 1A,B). The difference was especially evident for the lower mass range (*m/z* 380–700). Although extracted ion chromatograms were of similar quality (data not shown), interfering signals in CHAPS samples could contribute to lower resolving power and a decreased reliability of quantification. The effect of interfering signals was measured using signal-to-noise (S/N) ratio with peak detection ICIS algorithm using Xcalibur software. High abundant ions had similar S/N ratio for SDS and CHAPS samples but ions of lower abundance displayed higher variance. In particular, CHAPS treated samples presented lower S/N rations for low molecular oligosaccharide peaks with [M - H]^-^ions of *m/z* 464.5 (Hex_1_HexNAc_1_Sulfate_1_) (mean = 29), *m/z* 667.5 (Hex_1_HexNAc_2_Sulfate_1_) (mean = 151), and *m/z* 675.5 (NeuAc_1_Hex_1_HexNAc_1_) (mean = 140) than the SDS-treated samples (mean = 51; mean = 201; mean = 191, respectively). Moreover, to further optimize the method, we have tested different concentration of SDS in the solubilisation buffer (0.5%, 1% and 2%) and the resulting data were very similar (data not shown).

Since our protocol included an overnight solubilisation of the samples, at 4 °C with detergent and reducing agent, sonication did not prove to be of added benefit. The LC-MS profile of *O*-linked glycans is very similar with and without the sonication as shown in Fig. 1. Elimination of sonication step in the protocol speeds up the process without compromising on the quality of analyses.

Analysis of porcine gastric tissue samples using different conditions showed no major differences between the *O*-glycome. Therefore, to minimize variations and increase the speed and reproducibility of the analyses we decided to proceed with solubilisation buffer containing SDS and semi-automatic homogenization with Precellys system without sonication step.

### The *O*-glycans in human gastric tissue

**Figure 2:**
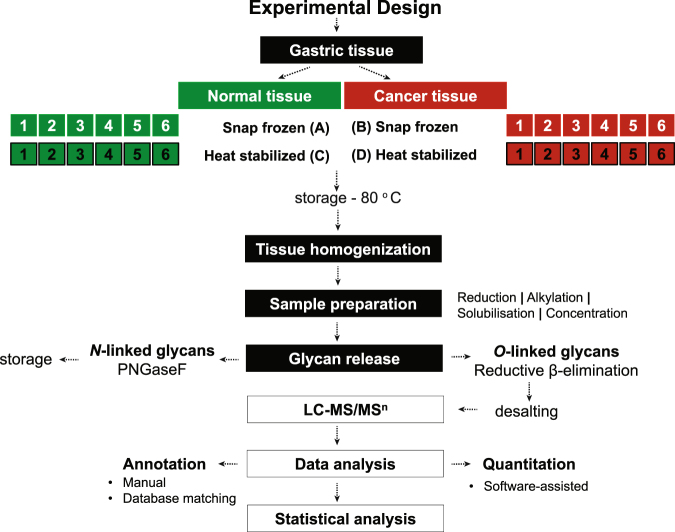
Overview of the workflow for *O*-glycan analysis from gastric tissue samples using LC-MS/MS.

**Figure 3:**
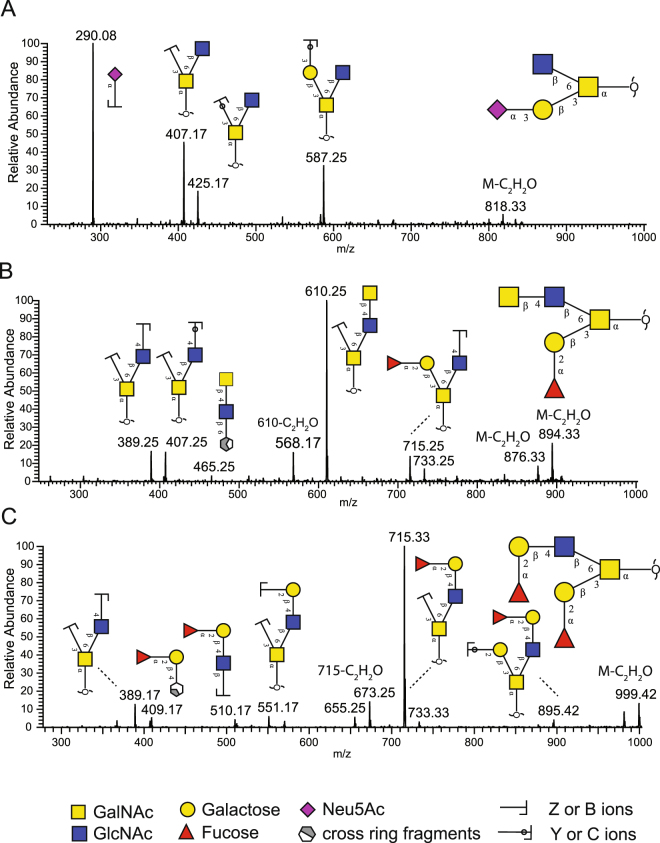
Examples of LC-MS/MS fragmentation spectra of glycan (**A**) sialylated structure with composition NeuAc_1_Hex_1_HexNAc_2_ [M-H]^−^ ion of *m/z* 878, (**B**) LacdiNAc containing *O*-glycan with composition dHex_1_Hex_1_HexNAc_3_ [M-H]^−^ ion of *m/z* 936. (**C**) Blood group H epitope containing glycan with composition dHex_2_Hex_2_HexNAc_2_ [M-H]^−^ ion of *m/z* 1041. Proposed structures are depicted using SNFG nomenclature^63^ and fragments of carbohydrates as defined by Domon and Costello^64^.

*O*-glycans from 6 individuals (Figure 2 and Table 1) were released from human gastric tissue by reductive β-elimination and were analyzed using LC-ESI-MS/MS. The resulting data was aligned, peaks picked and quantified with an aid of Progenesis QI software. All structures were manually annotated using monoisotopic precursor mass and CID-based MS/MS fragmentation. Intensities were converted to relative % abundance for every structure and log-transformed (Tables S1 and S2). In addition, several glycosylation features, based on the data and knowledge on carcinoma associated epitopes (Table S1), were derived from ratios of the glycan peaks, summing up all structures that contained the same structural trait, namely sialylation, sulfation, fucosylation, neutral glycans (Hex + HexNAc only), LacdiNAc (GalNAcβ1-4GlcNAc), and epitopes such as Lewis^a/x^, Lewis^b/y^, αGlcNAc, I branch, blood group H, A and B. Furthermore, mass spectrometry average compositions (Table S1) were calculated. Examples of fragmentation spectra and annotation for sialylated glycan with an [M – H]^−^ ion of *m/z* 878 (NeuAc_1_Hex_1_HexNAc_2_), LacdiNAc containing structure with an [M – H]^−^ ion of *m/z* 936 at 23.8 min (Hex_1_HexNAc_3_dHex_1_) and H blood epitope containing glycan with an [M – H]^−^ ion of *m/z* 1041 (Hex_2_HexNAc_2_dHex_2_) are represented in Fig. 3.

**Table 1:** Clinical characteristics of the patients.

**ID**	**Gender**	**Age**	**Blood Group**	**Stage**	**Diameter (mm)**	**Lauren**
1	Male	86	AB Rh+	IIIB	75	INT
2	Female	84	O Rh−	IIIC	40	MIX
3	Male	67	A Rh−	IIB	50	MIX
4	Male	85	O Rh+	IIIA	65	INT
5	Female	85	A Rh−	IIIB	60	INT
6	Female	91	A Rh+	IIB	55	INT

The resulting data confirmed the presence of at least 83 *O*-linked glycan structures and 17 derived features were examined to compare the *O*-glycosylation in human gastric tissue (Tables S1 and S2). Among identified structures, the majority was found to be core 2 *O*-glycans. Core 1, core 3 and core 5 were also present but they were of lower abundance (Table S1). The glycan chain length was between two and eleven monosaccharides.

### Sample handling post collection - snap frozen vs. heat stabilized

**Figure 4:**
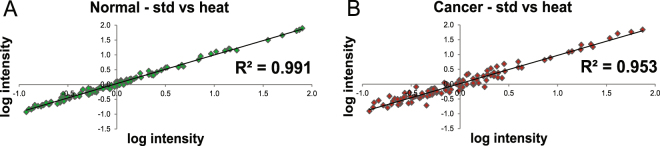
Correlation between samples using linear regression analysis (**A**) Normal samples – std vs heat stabilized, (**B**) Cancer samples – std vs. heat stabilized. These plots represent log-transformed data of the mean value for 88 variables for specified groups of samples.

A dataset of identified structures from four groups of tissue samples, normal - standard conditions (A), cancer - standard conditions (B), normal heat-stabilized post-collection (C) and cancer heat-stabilized post-collection (D) was generated as described in Fig. 2. No MS intensities from these structures were found to be statistically significant between normal standard (A) and normal heat-stabilized (C) samples as well as between cancer standard (B) and cancer heat-stabilized (D) (Table S2). In order to display this we generated the MS intensity correlation between different groups of samples using linear regression analysis. Coefficient of determination (denoted as R^2^) indicates how well all data points fall on the regression line. As shown on Fig. 4, variation between snap frozen and heat stabilized samples is very low, for normal samples R^2^ = 0.99 (Fig. 4A) and for cancer samples R^2^ = 0.95 (Fig. 4B). These data suggest that *O*-glycans are not influenced by the difference in stabilization methods. Notably, correlation between normal samples is higher than for cancer samples. It has been shown before^32^ and also our data suggest that cancerous *O*-glycans are more variable due to the different staging, local heterogeneity and histological manifestation of cancer. Hence, the lower R^2^ found in the cancer sample is probably reflecting a microheterogenetity between the paired cancer samples rather that a difference in the sample preservation method.

**Figure 5:**
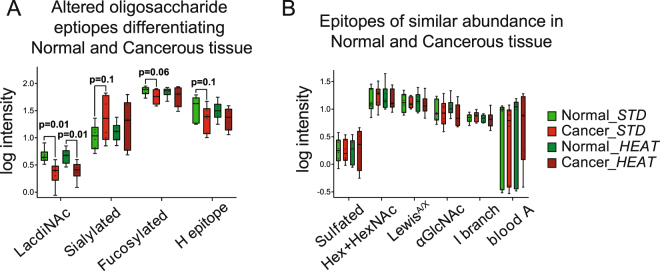
Boxplots representing distribution of log-transformed data (**A**) derived structural features that differed between normal and cancerous samples (**B**) derived structural features that were of similar abundance between normal and cancerous samples. Min to max value of derived features is plotted along with all points to show the distribution of variable, statistically significant difference is included as p value; also *p*-values below 0.1 are displayed showing trends in the data.

**Figure 6:**
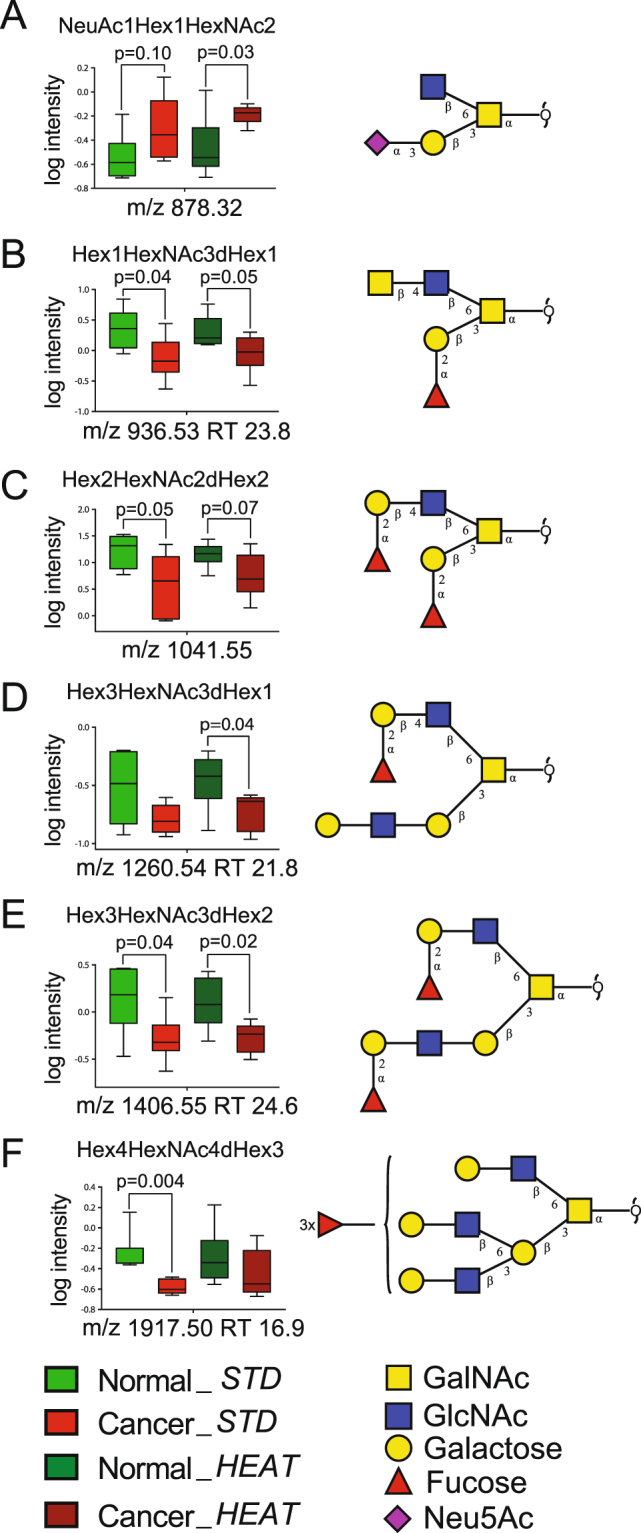
Change in glycosylation between normal and cancerous tissue samples. Only those box plots of glycan structures that were found to change significantly are shown here. (**A**) *m/z* 878, (**B**) *m/z* 936, (**C**) *m/z* 1041, (**D**) *m/z* 1260, (**E**) *m/z* 1406 and (**F**) *m/z* 1917. Data are given in a boxplot to show the spread of individual values. The diagram is made up of a ‘box’, which lies between the upper and lower quartiles. The median level divides the ‘box’ into two. The ‘whiskers’ extend from the ends of the box to the maximum and minimum values, respectively. Values for normal tissues are shown in green color (lighter for snap frozen samples, darker for heat-stabilized) and cancer tissues are shown in red (lighter for snap frozen samples, darker for heat-stabilized). The glycan structures are denoted using SNFG notation^63^. Statistically significant difference is included as p value; also *p*-values below 0.1 are displayed showing trends in the data.

In addition, we also examined differences in the structural features derived from the the identified structures. Box-plots displayed in Figs 5 and 6 confirmed that the *O*-glycans in snap-frozen and heat-stabilized samples was very similar. Hence, the conclusion is that the *O*-glycans did not differ between snap-frozen and heat-stabilized samples suggesting that heat stabilization is a good alternative for biobank preservation for glycomics and do not contribute to any unwanted degradation.

### Differences between normal and cancerous tissue *O*-glycome

The subsequent phase of the study was aimed at identification of *O*-glycans that were altered between normal and cancerous tissue. Statistical analysis was carried out in order to detect any changes in glycosylation. A returned *p* value less than 0.05 was used as a first screen and relevance of observed alterations was further examined using box-plots. In total, 9 out of the 83 *O*-glycan structures and 1 out of 17 calculated structural features were returned as being altered for normal and cancerous samples (Table S1). In addition, 8 glycan structures and 3 calculated features reached a *p* value of 0.1 and showed decreasing or increasing trend between sample classifications. To evaluate the trends further, box-plots were generated. Figure 5A displays results for log-transformed sum of the derived features that were different between normal and cancerous samples. The most significant difference can be seen for the LacdiNAc epitope (*p* = 0.013 for AB groups (snap frozen) and *p* = 0.015 for CD groups (heat stabilized). Of note from Fig. 5A is also an increasing trend for sialylated structures in cancerous tissue (*p* = 0.1) and a decreasing trend for fucosylated structures and H type epitope containing structures (*p* = 0.06 and *p* = 0.10, respectively). Also of note is the distribution within each box-plot, tight for normal samples and with higher spread for cancerous samples, which can indicate higher diversity within different subtypes or stages of gastric cancer. Similar trends between normal and cancerous samples are seen for levels of mass spectrometry average composition (Table S1).

Analysis of the individual glycan structures showed several alterations in glycosylation between normal and cancerous samples. Nine glycans out of 83 had *p* value less than 0.05 and six with the lowest *p* value are depicted in Fig. 6. Alterations were identified in structures containing sialic acid, blood H-type epitope and LacdiNAc. Specifically, the levels of *m/z* 878 (NeuAcα2,3Galβ1,3(GlcNAcβ1,6)GalNAcol) were higher in cancerous samples (0.27 ± 0.07%, mean ± SEM in normal samples to 0.54 ± 0.09% in cancerous tissues) (Fig. 6A). Similarly, the levels of *m/z* 675 (Galβ1,3(NeuAcα2,6)GalNAcol and NeuAcα2,3Galβ1,3GalNAcol), *m/z* 966 (NeuAcα2,3Galβ1,3(NeuAcα2,6)GalNAcol), and *m/z* 1331 (NeuAcα2,3Galβ1,3(NeuAcα2,3Galβ1,4GlcNAcβ1,6)GalNAcol) were also increased in cancerous samples, although with a p > 0.1 (1.40 ± 0.19%, mean ± SEM in normal samples to 3.35 ± 0.93% in cancerous samples; 2.75 ± 0.42% in normal samples to 6.57 ± 1.83% in cancerous samples; 1.70 ± 0.20% in normal samples to 5.69 ± 1.91% in cancerous samples; 0.51 ± 0.05% in normal samples to 3.35 ± 1.24% in cancerous samples, respectively).

Interestingly, a decrease in a biantennary structure containing both blood group H as well as a LacdiNAc epitope with an [M – H] of *m/z* 936 (Fucα1,2Galβ1,3(GalNAcβ1,4GlcNAcβ1,6)GalNAcol) was also observed (Fig. 6B); higher levels of this structure were present in normal samples than those in the cancerous condition (2.64 ± 0.60%, mean ± SEM vs. 0.97 ± 0.23%, mean ± SEM). In addition to *m/z* 936, two more structures have been identified to contain LacdiNAc eptiope, *m/z* 790 (Galβ1,3(GalNAcβ1,4GlcNAcβ1,6)GalNAcol) and *m/z* 1139 (GalNAcα1,3(Fucα1,2)Galβ1,3(GalNAcβ1,4GlcNAcβ1,6)GalNAcol). Both, were of higher abundance in normal tissues in comparison to cancerous samples (1.34 ± 0.26%, mean ± SEM in normal vs. 0.97 ± 0.14%, mean ± SEM in cancer, 0.76 ± 0.18%, mean ± SEM in normal vs. 0.51 ± 0.15%, mean ± SEM in cancer, respectively).

Four glycan structures ([M – H]^−^ ions of *m/z* 1041 (Hex_2_HexNAc_2_dHex_2_), *m/z* 1260 (Hex_3_HexNAc_3_dHex_1_), *m/z* 1406 (Hex_3_HexNAc_3_dHex_2_), and *m/z* 1917 (Hex_4_HexNAc_4_dHex_3_)) were decreased (p < 0.1 in AB and/or CD set) in cancerous tissue samples (Fig. 6C–F and Table S2). All of them contain blood H type epitope, α1,2 fucose residue.

Intrigued by all the trends in the change of glycosylation between healthy and cancerous tissue seen already by analyzing only six individuals, additional samples and analysis will be required for verification of the findings. Stringent statistical evaluation requires a larger clinical sample set when a large number of variables (n = 100 (83 structures +17 derived features)) are evaluated. This is illustrated in our data were the significance levels of the paired sample t-tests on glycosylation structures and features were further corrected for multiple comparison and adjusted p-values (q-values) increased (Tables S1 and 2). In all figures, we present non-adjusted p-value to show only the trends in the data.

## Discussion

This is the first study to investigate the effect of heat-stabilization treatment on *O*-glycosylation in human gastric tissue. Tissue samples were obtained from patients undergoing gastric cancer surgery (n = 6), for each individual a pair of non-tumorigenic and tumorigenic samples were collected to minimize individual differences due to age, smoking, body mass index (BMI), medication and other factors which have been reported previously to affect the glycosylation of proteins^33,34^. Each sample was further divided into two fractions post-sampling, one was immediately snap-frozen and second one heat-stabilized post-collection and then placed in −80 °C freezer. This experimental setup allowed for addressing two aims, a) effect of sample preservation technology on *O*-glycome and b) identification of characteristics differentiating tumor tissue from non-tumor tissue that might lead to clinical tests for detecting, monitoring or treatment of gastric cancer.

Within the field of glycan analysis, sample preparation is often an overlooked but highly important step of procedure. We have optimized the protocol to ensure efficient extraction of glycoproteins from tissue samples using a semi-automatic system. There are many detergents that are currently used in protein solubilisation, including anionic and zwitterionic detergents (SDS and CHAPS, respectively) among others. SDS is a routinely used reagent for maximum protein solubility and was shown previously to be a superior detergent for extraction of proteins during homogenization compared to ASB-13, SB3-10 or CHAPS^35^. However, due to its charged nature it is incompatible with some analyses such as isoelectric focusing (IEF) or acidic protein enrichment. Another important consideration for selecting a detergent is the possibility to remove it before analysis as detergents can be problematic for mass spectrometry. Inability to remove these substances might result in a high chemical background and deterioration of the mass spectrometers behavior. Extensive washing needs to be performed prior to glycan release as well as additional clean-up prior to LC-MS separation. However, as we noted during sample optimization, the glycan distribution was very similar for SDS and CHAPS-treated samples but interfering signals were higher for CHAPS. We also observed that sonication had no effect on abundance of *O*-glycans extracted from porcine gastric tissue, so this step was excluded in the analysis of the 24 human samples.

An important strength of the optimized method is the semi-automatic homogenization of tissue samples, performed for 24 samples simultaneously. We tested manual homogenization versus a higher throughput homogenization with ceramic beads and observed that both provide comparable results. Sample homogenization with bead-based technology appears to be a good alternative to the manual homogenization as it provides an increased speed and reproducibility of extraction procedures. Recently, several high-throughput workflows have been published for large-scale glycoprofiling^36–40^. These methods were shown to be well-suited for glycan analysis of plasma, serum or purified glycoproteins, allowing for automation in the 96-well format. However, they were developed for liquid-based matrices that do not require time-consuming homogenization of tissue samples.

Our data showed that *O*-glycosylation of snap frozen and heat-stabilized samples did not differ. The relative abundance of sialylation and sulfation but also other structural features remained the same with the different treatments. The data suggest that heat stabilization does not provide any advantage over standard operating procedures currently employed at the biobanking facility in Siena. However, sample quality can deteriorate rapidly, especially if the specimen undergoes multiple thaw/freeze cycles or aliquoting^41^. In our dataset, there was no pre-analytical variability related to processing delays. All samples were collected in a short time, snap frozen or heat-stabilized immediately post-excision and placed in −80 °C freezer. It has been demonstrated previously that even a 30 min delay before snap freezing can contribute to a significant decrease in the phosphorylation states^42^. Enzymatic activity is stopped when samples are frozen but once the temperature is raised, the enzymes resume their activity and can contribute to unwanted degradation. We could not test what effect thawing/freezing can have on glycosylation but in the long term perspective, there is potentially an advantage of using heat-stabilization over snap freezing. Heat stabilization inactivates enzymes permanently whereas snap freezing only temporarily. Our study analyzed effect of heat stabilization vs. snap freezing on *O*-glycosylation. However, it would be interesting to test stabilization platform also for other post translational modifications. Heat stabilization has been shown to provide superior results over snap freezing for phosphorylated proteins and endogenous peptides^43,44^. Although *O*-glycosylation profiles appeared to be the same between snap frozen and heat-stabilized tissue, tissue stabilization offers the potential to better preserve tissue for other downstream processing and reduce the variability related to processing delays.

Alterations in gastric cancer protein glycosylation were reported previously in *N-* and *O*-glycosylation pathways. The majority of glycomic studies performed to date have focused on *N*-glycans on plasma or serum samples^45,46^ but also on purified glycoproteins such as IgG or E-cadherin^47,48^. The most prominent alterations are seen in *N*-glycan branching and increase in sialylation and fucosylation. Another common feature observed in gastrointestinal tumors is the overexpression of short, truncated *O*-glycans (e.g. Tn, sTn and T antigens) and increased sialylation of complex structures (e.g. sLe^a^ and sLe^x^ antigens) on mucin type *O*-glycans^49,50^. However, our experimental setup did not allow detecting the Tn-antigen monosaccharide.

High level of structural diversity of human and porcine gastric mucin glycans was reported previously, both within individuals and different layers of stomach and regions^51,52^.

In the current study, comparison of paired tumor and non-tumor tissue samples revealed a number of alterations present in *O*-glycan structures and derived features. The levels of LacdiNAc epitope were significantly reduced in cancerous samples. Interestingly, this epitope was previously found on a secreted MUC5AC on gastric mucosa and Kenny *et al*. showed that it was a dominating structure in gastric normal tissue and possibly protecting against *H. pylori* binding^53–55^. In addition, the mean level of % of sialylated structures, as well as individual sialylated structures ([M – H]−ions of *m/z* 675, 878, 966, and 1331) were shown to be increased in cancerous samples. This finding is in agreement with previous studies of gastric cancer^5^. Among significant structures, four glycans were significantly decreased in cancerous tissue. The common structural characteristics for all these samples was blood H-type epitope, α1,2 fucose. This epitope has been previously suggested to inhibit vasculogenesis and tumor growth in hepatocarcinoma cells^56^.

There has always been a trade-off between the sensitivity of fine structure characterization of glycan mixture and the ability to perform a high-throughput study of a large number of samples. Paired analysis of tumor and neighboring normal gastric mucosa is a valuable methodology employed to identify those changes that can be associated with tumorigenesis. Our experimental setup included samples from six individuals that were divided into four groups representing different treatment post-collection which allowed us to address two questions a) effect of heat stabilization vs. snap freezing of tissue samples post excision and b) alterations between normal and cancerous mucosa. The significance of reported alterations for gastric cancer needs to be investigated further on a larger cohort. In addition, the heterogeneity of especially the carcinoma tissue indicates that the glycosylation changes need to be addressed on subsection based on pathological scoring of tissue section. The *O*-glycan alterations that are presented here may provide predictive power for the detection and prognosis of gastric cancer. Further studies are necessary to determine their clinical potential for improving the accuracy of stomach biopsy diagnostics performed in connection to endoscopic examination.

## Conclusions

This study shows that there is no significant difference of the gastric *O*-linked glycans released from post excision thermal stabilized tissue and snap frozen tissue. The data also reveal that cancer tissues display different glycosylation to the adjacent normal tissues. Specifically, tumorigenic tissues appeared to show an increase in sialylation and a decrease in LacdiNAc and blood H-type epitope containing glycan structures. As glycosylation impacts on many important cellular processes including cellular recognition, protein function, protein stability and immunogenicity, cancer-induced glycosylation changes may trigger a number of downstream effects.

## Materials and Methods

### Study subjects and clinical description

The study cohort consisted of 6 patients that undergone gastric cancer surgery at the Department of General Surgery and Surgical Oncology, University of Siena, Siena, Italy. The characteristics of the study group are listed in Table 1. All six individuals were recruited locally, 3 females and 3 males. The age range for entire cohort was 67–91 years with median age 85. The study conformed to the ethical guidelines of the 1975 Declaration of Helsinki and experimental protocols were approved by Comitato Etico Regione Toscana. Methods were carried out in accordance with approved protocols with all participants signing informed consent prior to participation.

Table 1. Gastric Cancer Patient Clinical Characteristics, stage is indicated using the American Joint Committee on Cancer (AJCC) classification, tumor diameter is in mm and the histological classification is based on Lauren’s criteria.

### Tissue Collecting Conditions

All subjects underwent a surgical procedure with a complete resection of the tumor. Depending of the tumor location, distal subtotal gastrectomy was performed or a total gastrectomy as described in^57,58^. Four tissue specimens were collected from each subject (total n = 24), two samples of tumor mucosa and two from adjacent normal mucosa. Collected samples were either snap-frozen in liquid nitrogen (n = 12) or heated at 95 °C in the Stabilizor T1 instrument (Denator AB, Uppsala, Sweden) at auto settings for fresh tissue (n = 12)^10^ (Fig. 2). All samples were then transferred to −80 °C until further analysis.

### Chemicals and Reagents

Reagent water used throughout this study was obtained from an Elga LabWater PureLab flex system (Veolia Water Technologies, High Wycombe, UK) and was 18.2 MΩ or greater with a total organic carbon content less than 5 parts per billion. Acetonitrile, methanol and water was of LC-MS grade and sodium hydrogen carbonate (NaHCO_3_) was attained from Merck-Millipore (Darmstadt, Germany). Acetic acid, thiourea and sodium dodecyl sulfate (SDS) were from VWR (Radnor, PA, USA). Ammonium bicarbonate ((NH_4_)HCO_3_), sodium borohydride (NaBH_4_), sodium hydroxide (NaOH), tris, urea, dithiothreitol (DTT), as well as iodoacetamide (IAA), was from Sigma-Aldrich (St. Louis, MO, USA).

### Sample Homogenization

For optimization of homogenization protocol we have used porcine gastric tissue and several conditions were tested. In particular, manual homogenization with sonication was compared with automatic homogenization using Precellys system (Bertin Technologies, Montigny-le Bretonneux, France). For the extraction of proteins from porcine gastric tissue, 50 mg (wet weight) sample tissue was weighted, 1 mL of solubilisation buffer was added to the tube (7 M urea, 2 M thiourea, 40 mM Tris and a protease inhibitor cocktail at the dilution 1:100 (Sigma-Aldrich)) and the tissue was homogenized using a VDI-12 homogenizer (VWR), 4 × 15 sec, or using a Precellys 24, 2 × 30 sec cycles, samples were kept on ice between the cycles. Homogenization was followed by sonication (MSE Soniprep 150, 4 × 15 sek, 16 micron amplitude setting) at 4 °C for one batch of the samples but for second batch sonication was not performed to verify if this step was essential for efficient tissue homogenization. Further, different concentrations of detergent have been tested (0.5%, 1% and 2%). After homogenization, detergent (CHAPS or SDS) and 10 mM DTT were added to the samples to facilitate solubilisation. Following overnight incubation at 4 °C on a rotor, 25 mM IAA was added and incubated at RT in the dark for 40 min. Glycan release, desalting and LC-MS analysis of *O*-linked glycans was performed using the same protocol (as described in the subsequent sections) for all the samples.

The snap-frozen and heat stabilized human tissue samples were weighted (50 mg, wet weight) into 2 mL reinforced homogenization tubes (Bertin Technologies) and loaded with 6 zirconium oxide beads (3 mm) from Bertin Technologies. During weighing, the samples were kept on ice to ensure temperature control during sample handling. The tissue was then homogenized in 500 μL solubilisation buffer (7 M urea, 2 M thiourea, 40 mM Tris and a protease inhibitor cocktail at the dilution 1:100 (, Sigma-Aldrich)) at power 5500 using a Precellys 24, 2 repeated 30-second cycles. Following the homogenization, another 500 μL of solubilisation buffer was added including SDS and DTT (final concentration 0.5% SDS and 10 mM DTT). The extraction of up to 2 × 24 tubes was then continued at 4 °C overnight on a rotor. After overnight solubilisation, 25 mM IAA was added and incubated for 40 min at RT in the dark. The tissue extracts were centrifuged for 20 min at 13,200 rpm and the supernatants were transferred to a new tube and stored at −20 °C until further analysis.

### Glycan Release and Desalting

Tissue extracts (100 μL) were diluted 10 × with buffer (20 mM NaHCO_3_) and applied to a preconditioned 10 kDa cut-off filter (Pall, Port Washington, NY, USA). Samples were centrifuged for 10 min at 13,200 rpm, 2 × 500 μL and washed 3 times with 200 μL 20 mM NaHCO_3_. *N*-linked glycans were released by incubation using 50 μL 20 mM NaHCO_3_ and PNGaseF (5mU, Prozyme, Hayward, CA) at 37 °C overnight. Subsequently, *N*-glycans were collected by washing 3 times with 50 μL H_2_O and dried in SpeedVac. The *O*-glycans were then released from retained glycoproteins in filters by incubation with 50 μL 0.5 M NaBH_4_ in 50 mM NaOH, 16 hr, at 50 °C^59^. The reductive β-elimination reaction was quenched by addition of 2.5 μL of acetic acid and *O*-glycans were eluted with 2 × 100 μL H_2_O and then desalted with 35 μL of AG50WX8 cation exchange slurry (Bio-Rad, Hercules, CA) packed on the top of C18 zip tips (Millipore). The samples were then dried in the SpeedVac and borate complexes were removed by repeated addition/evaporation of 50 μL of methanol (five times). The released oligosaccharides were dissolved in water for LC-MS/MS analysis.

### LC- MS/MS of *O*-linked glycans

Released *O*-glycans were analyzed by LC-ESI-MS/MS using a 10 cm × 250 μm I.D. capillary, prepared in-house, containing 5 μm porous graphitized carbon (PGC) particles (Thermo Scientific, Waltham, MA). Solvent A was 10 mM (NH_4_)HCO_3_ and solvent B was 10 mM (NH_4_)HCO_3_ in 80% acetonitrile. Glycans were eluted using a linear gradient from 0 to 45% buffer B over 45 min at a flow rate of 10 μL/min. The eluted *O*-glycans were detected using a LTQ ion trap mass spectrometer (Thermo Scientific) in negative-ion mode with an electrospray voltage of 3.5 kV, capillary voltage of −33.0 V and capillary temperature of 300 °C. Air was used as a sheath gas and a full scan ranges from *m/z* 380 to *m/z* 2000 were defined for the structures to be analyzed, selecting 9 most intense ions from the full scan with an intensity of 300 and dynamic exclusion for 2 min.

### Data Analysis

The *O*-glycan structures were characterized using accurate monoisotopic precursor mass, PGC-LC retention time and collision-induced dissociation (CID)-based MS/MS fragmentation. The data were processed using the Xcalibur software (version 2.2, Thermo Scientific) and manually interpreted from their MS/MS spectra. The Thermo RAW files were loaded into the Progenesis QI software (Nonlinear Dynamics, Newcastle, UK), aligned using automatic alignment and the samples were grouped according to experimental conditions (A - normal, STD, B - cancer, STD, C - normal, HEAT, D - cancer, HEAT) using within-subject design. Peak picking parameters were set automatic sensitivity with a default noise estimation algorithm and retention time limits 7 to 30 min. Compounds without MS/MS spectra were deleted from the analysis, as this information in required for structure identification. The compounds were searched against the reference library (developed in-house) and downloadable from the UniCarbDB database (http://unicarb-db.expasy.org/)^60,61^. All compounds were manually verified using RT and MS/MS fragmentation. Structures and spectra will be available in next update of UniCarbDB at (http://unicarb-db.expasy.org/references/344). Resulting table with compounds of interest and raw abundance was exported to an Excel file for further analysis. In addition to individual structures, several derived featrues were calculated. Level of sialylation, sulfation, fucosylation, neutral glycans (Hex and HexNAc),LacdiNAc (GalNAcβ1-4GlcNAc), epitopes such as Lewis^a/x^, Lewis^b/y^, αGlcNAc, I branch, blood group H, A and B were calculated by summing up all structures that contained the same structural trait. Furthermore, mass spectrometry average compositions were calculated as described previously^62^. Briefly, all structures were reduced to monosaccharide compositions and were multiplied by percentage intensity for each structure and summed over the entire sample.

### Statistics

The relative abundance of *O*-glycan structures is compositional by nature, since the data convey the relative percentage areas from the overall *O*-glycome LC-MS profile rather than absolute quantities. Therefore, the data were log-transformed (log(x + 0.1)) to satisfy parametric test assumptions. Paired t-tests were carried out to determine if there were significant differences between the groups. Significance levels of t-tests were evaluated with a cutoff p-value of 0.05 for the glycan structures and derived glycosylation features and were corrected for multiple comparisons using the Benjamini-Hochberg adjustment. Analysis was performed using the R software (www.r-project.org) with the R base package and boxplots were constructed using GraphPad Prism 7 software (San Francisco, CA, USA).

### Data availability

Unprocessed MS files for all the samples are available from local web location according to Table S3. MS/MS spectra representative for each assigned structure in Table S2 is available at http://unicarb-db.expasy.org/references/344).

## Electronic supplementary material

Supplementary Dataset 1

Supplementary Dataset 2

Supplementary Dataset 3
